# A simple way to treat penile concealing due to webbed penis

**DOI:** 10.1590/S1677-5538.IBJU.2019.0798

**Published:** 2020-11-18

**Authors:** Antonio Macedo, Sergio Leite Ottoni, Gilmar Garrone, Ricardo Marcondes de Mattos, Marcela Leal da Cruz

**Affiliations:** 1 CACAU-NUPEP Departamento de Urologia São PauloSP Brasil Departamento de Urologia, CACAU-NUPEP, São Paulo, SP, Brasil; 2 Universidade Federal de São Paulo Departamento de Pediatria São PauloSP Brasil Departamento de Pediatria, Universidade Federal de São Paulo, São Paulo, SP, Brasil

## Abstract

**Introduction:**

The webbed penis represents a common genital abnormality consisting of penoscrotal transposition of various degrees, the presence of a skin fold tethering the ventral penile shaft to the scrotum promoting the absence of a penoscrotal angle and an abnormally short ventral shaft. Besides, a stenotic ring of distal prepuce (phimosis or paraphimosis) is frequently found. We want in this video to illustrate the steps of this common procedure associated with an excellent cosmetic result and improvement of self-esteem.

**Patients and Methods:**

Surgery consists of treating penoscrotal transposition when present by two inverted scrotal V-shaped skin flaps to be brought down to its natural position. The ventral penile shaft is detached from the scrotum, excising or dividing the fibrotic and fatty tissue. We dissect the skin and deglove the penis proximally almost reaching the pelvic floor, producing a release of the penile shaft and increase in size. After that, we suture the ventral penile skin at the lowest level of dissection by two 3.0 vycril sutures anchoring them to the Buck's fascia one at each side of the urethra. Subsequently, the circumcision is performed and the scrotum reconstructed with removal of redundant skin when necessary.

**Results:**

Surgery produced improvement of ventral surface of the penis and better cosmetic appearance without any local complication

**Conclusion:**

The webbed penis is a frequently under-recognized abnormality by pediatricians, but a major cause of anxiety for parents. This technique can be regarded as an alternative to most webbed penis patients.

